# Enhanced Electrorheological Response of Cellulose: A Double Effect of Modification by Urea-Terminated Silane

**DOI:** 10.3390/polym10080867

**Published:** 2018-08-04

**Authors:** Zhao Liu, Panpan Chen, Xiao Jin, Li-Min Wang, Ying Dan Liu, Hyoung Jin Choi

**Affiliations:** 1 State Key Lab of Metastable Materials Science and Technology, College of Materials Science and Engineering, Yanshan University, Qinhuangdao 066004, China; liuzhao0120@sina.cn (Z.L.); 15032375221@139.com (P.C.); jinxiao94315@163.com (X.J.); limin_wang@ysu.edu.cn (L.-M.W.); 2 Department of Polymer Science and Engineering, Inha University, Incheon 402751, Korea

**Keywords:** electrorheological fluid, cellulose, swelling, silane, yield stress, dielectric spectra

## Abstract

As a natural polymer with abundant sources, cellulose was one of the earliest applied electrorheological (ER) materials. However, cellulose-based ER materials have not attracted much attention because of their relatively low ER effect and sensitivity to water. In this study, cellulose rods were decorated with a urea-terminated silane, 1-(3-(trimethoxysilyl) propyl) urea, after being swelled in sodium hydroxide solution. The morphologies and structures of the cellulose particles were investigated using scanning electron microscopy, Fourier-transform infrared spectroscopy and X-ray diffraction, confirming the dramatic differences of the treated cellulose particles from the pristine cellulose. Rheological behaviors of the pristine and modified cellulose particles in silicone oil were observed using a rotational rheometer. It was found that the silane-modified cellulose showed higher ER effect and higher dielectric properties than the pristine cellulose particles, which was not only related to the grafted polar molecules but may also be associated with the porous morphologies of the treated cellulose particles.

## 1. Introduction

Cellulose is the most abundant biopolymer, and comes from the cell wall of plants and bacteria, having an annual production of 1.5 × 10^12^ tons [1,2]. The current environmental and energy-related problems have been motivating the development of novel production approaches that are both sustainable and eco-friendly. As a renewable, biodegradable and inexpensive material, cellulose has attracted much attention since it was initially characterized in 1838 [3]. It is well known that cellulose is composed of three basic elements of C, H and O, being a typical carbohydrate. d-pyranose, which has three –OH groups as the repeating units linked by the glycosidic bond, forms the chain conformation with successive glucose rotated through an angle of 180° about the molecular axis and hydroxyl groups in an equatorial position [4,5,6,7]. The physical and chemical properties of cellulose are different from those of other polymers and are largely dependent on its supramolecular structure [8,9,10,11]. The supramolecular structure of cellulose is not simply based on the gathering of the six-membered rings held together by physical interaction [12]. Rather, the anhydroglucose units (AGUs) are linked by β-1,4-glycosidic linkages, a kind of covalent bond, considered to be the specific structure. The large cohesive energy in cellulose obviously reveals extensive hydrogen networks between intermolecular and intramolecular interactions formed by three –OH groups [13,14]. The ability of these hydroxyl groups to form hydrogen bonds plays a major role in the formation of fibrillar and semicrystalline packing, hence cellulose has at least five allomorphic forms [1,15,16,17]. Both physical and chemical methods can be used to change the morphology and properties of cellulose. A high-pressure homogenization method [18,19] and ball-milling [20,21] are used to make the cellulose smaller and amorphous. Noncovalent modification [22], sulfonation [23], TEMPO-mediated oxidation [24], esterification [25,26,27], etherification [28] and silylation [29,30] are chemical approaches applied to change the properties of cellulose. These effective methods allow cellulose to have more extensive applications, such as in composite materials, optical films, pharmaceuticals and foodstuffs, as well as in the dispersed phase of electrorheological fluids [26,27,31]. 

Electrorheological (ER) fluids are a form of smart colloidal suspensions, whose rheological properties will change significantly upon the application of an electric field [32,33,34]. The ER fluids consist of dielectric or semiconducting particles as the dispersed phase and insulating liquids as the continuous phase. When an electrical field stimulus is applied, the dispersed particles are able to be polarized and form clusters, chains or column structures. This makes the ER fluids transform from a liquid-like state to a solid-like state possessing a typical yield stress. The rheological properties of an ER fluid, including flow curves of shear stress or shear viscosity and dynamic modulus, are mainly dependent on the field-induced structures formed by the dispersed particles. To date, a variety of inorganic and organic materials that have specific ER effects when dispersed in insulating liquids have been investigated. Some compounds are attracting attention, such as titanium oxide with different morphologies, and calcium and strontium titanates, because of their high dielectric polarizability. The yield stress of these kinds of inorganic-based ER fluids can be over 130 kPa or higher when modified by polar molecules [35,36,37,38]. The high yield stress is actually enough for applications, however, inorganics always result in serious abrasion to devices and have poor suspension stability. Polymer-based ER materials are attractive candidates as they can overcome some drawbacks of inorganics, but they have relatively lower ER effects. Chemical modification and composite methods have been applied to enhance the ER effects of polymer-based ER materials [39,40,41,42]. 

Cellulose particles, as well as other biopolymers such as starch and chitosan, have been applied as ER materials because of their electro-responsive polar groups [43,44,45]. However, the pristine forms of these biopolymers are very sensitive to water due to their hydroxyl groups, which reduces the thermal stability and long-term stability of the ER fluid. Therefore, anhydrous biopolymers were introduced via various chemical modification methods including phosphorate and carbamate esterification. Consequently, outstanding ER effects have not been observed from these cellulose esters and microcrystalline cellulose particles. In the current study, urea-terminated silane was applied to modify cellulose particles via a hydrolysis process [46], before which the cellulose particles were swelled in sodium hydroxide solution. It was found that the chemically treated cellulose particles were extremely different from pristine cellulose in both morphology and structure. Rheological analysis indicated that the modified cellulose showed higher ER effect, which was attributed to the porous morphologies and decorated urea groups caused by the chemical modification process. 

## 2. Materials and Methods

### 2.1. Materials

Cellulose powders (C6288) used in this study were purchased from Sigma Aldrich (Shanghai, China). 1-(3-(trimethoxysilyl) propyl) urea (T1915, TCI, Shanghai, China), silicone oil (viscosity: 50 cSt; density: 0.96 g/mL) (Beijing Hangping Guichuang Chemical Co., Ltd., Beijing, China), sodium hydroxide (NaOH, analytical purity) (Tianjin Kaitong Chemical Reagent Co., Ltd., Tianjin, China), and hydrochloric acid (HCl, analytical purity) (Tianjin Jindongtianzheng Chemical Reagent, Tianjin, China) were used without any further purification. 

### 2.2. Preparation of Silane-Modified Cellulose (Cel-Si-Urea)

**Scheme 1:**
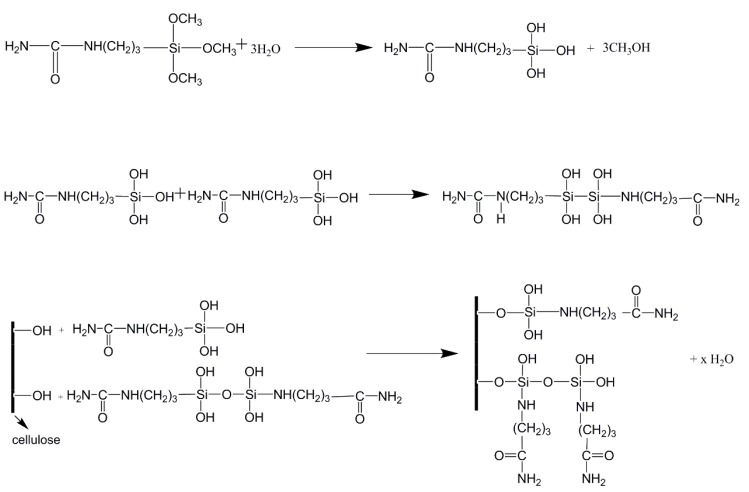
Schematic interaction process of silane with cellulose particles via a hydrolysis process.

5.0 g of cellulose powders were firstly dispersed in an aqueous solution of NaOH (10 wt %) and the suspension was precooled to −13 °C in a refrigerator to make the cellulose particles swell in the alkali solution. Then, the suspension was stirred vigorously until the suspension became semitransparent. 0.1 mol/L HCl solution was dropped into the above suspension until the pH value reached 7.0. In this process the gelatinous cellulose precipitated and the suspension became opaque white. Another liquid mixture was then prepared by adding 40 mL alcohol to 160 mL of deionized water, into which the cellulose suspension was poured. 1.0 g of 1-(3-(trimethoxysilyl) propyl) urea, pre-hydrolyzed in a small amount of water, was then dropped into the cellulose suspension. The suspension was stirred at room temperature for 4 h, then the precipitates were washed with deionized water several times and freeze-dried. A schematic reaction process between the cellulose and silane molecules is shown in Scheme 1.

### 2.3. Characterization

Fourier-transform infrared spectroscopy (FT–IR) (E55+FRA106, Bruker, Karlsruhe, Germany) was applied to examine the chemical structures of the cellulose after modification. Morphologies of the cellulose particles before and after treatment were observed by scanning electron microscopy (SEM) (S4800, Hitachi, Tokyo, Japan). The crystal structures of the cellulose were characterized using a powder X-ray diffraction pattern (XRD) (MAX-2500PC, Rigaku, Tokyo, Japan) with a Cu-Kα radiation source. The thermal stability of the samples was determined using a thermogravimetric analyzer (TGA) (STA4993, Netzsch, Selb, Germany) with a heating rate of 10 °C/min in the temperature range of 25 to 800 °C in air atmosphere. 

### 2.4. Preparation of ER Fluids and Rheological Measurements 

**Scheme 2:**
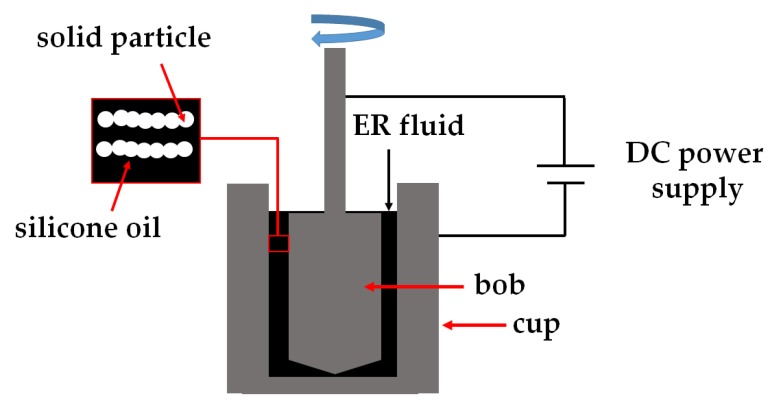
Schematic sectional diagram of the concentric cylinder (CC) geometry connected with a DC power supply. The electrorheological (ER) fluid is filled in the gap of the CC geometry.

Two ER fluids were prepared using pristine and modified cellulose (Cel-Si-Urea) particles, respectively. The particles were dispersed in silicone oil and treated by ultrasonication to form a uniform suspension, with a mass fraction of 15%. The rheological properties of the ER fluids were measured using a rotational rheometer (MCR502, Anton Paar, Graz, Austria) with a concentric cylinder (CC) geometry and a DC power supply. A schematic sectional diagram of the CC geometry composed of a bob and a cup is shown in Scheme 2. In the measurement state, the bob was dipped into the cup at a fixed position. The ER fluid was filled in the gap of the bob and the cup (gap distance = 0.7 mm). When the DC power supply was turned on, an electric field formed between the bob and the cup, and then the particulate chain-like structures formed in the direction of the electric field. If the bob was rotated, the torque (*M*) from the ER fluid could be sensed and measured by the rheometer. In the steady shear mode, the shear rate ($\dot{\gamma })$ of the bob was set in the range of 0.01–1000 1/s. The shear stress (*τ*) and shear viscosity (*η*) were calculated using the measurement software using the equations as follows,

(1) $$\tau =\frac{1+\delta^{2}}{2\delta^{2}}\cdot \frac{M}{2\pi L\cdot r_{i}^{2}\cdot C_{L}},$$

(2) $$\eta =\frac{\tau }{\dot{\gamma }},$$

(3) $$\dot{r}=\omega \cdot \frac{1+\delta^{2}}{\delta^{2}-1},$$

where *δ* = *r_e_*/*r_i_* represents the radius ratio of the cup (*r_e_*) and the bob (*r_i_*), *L* is the gap length of the measurement system, *ω* represents the angular velocity, and *C_L_* is an end-effect correction factor. Oscillatory strain sweep was performed in the strain range of 0.001%–10% at a constant frequency of 10 rad/s. The frequency sweep was performed in the frequency range of 1–100 rad/s with a constant strain of 0.003%. For all the measurement processes, the electric field strength was set individually by the measurement software. 

## 3. Results and Discussion

### 3.1. Morphologies and Structures

**Figure 1:**
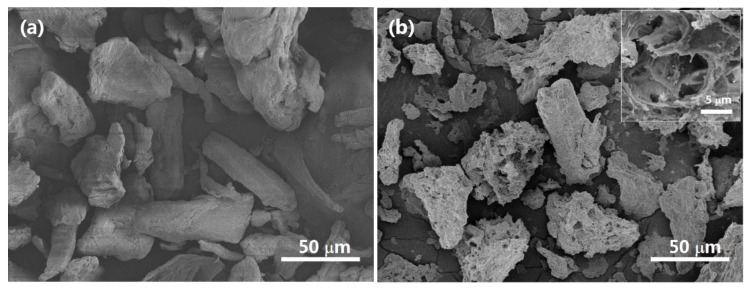
SEM images of the pristine (**a**) and urea-terminated silane-modified (**b**) cellulose particles.

The morphologies of the cellulose particles, including particulate shape, size and surface condition, were observed by SEM and are shown in Figure 1. The pristine cellulose particles (Figure 1a) had irregular shapes amongst which some were rod-like with particle sizes ranging from 20 to 100 μm. Particles treated in NaOH solution and modified by urea-terminated silane (Figure 1b) showed a similar size but became porous with pore sizes of 5–20 μm, which can be seen clearly from the inset of Figure 1b. Consequently, the surfaces of the particles become rougher than the pristine cellulose particles. These morphological characteristics of the treated cellulose either relate to the low-temperature swelling process in the NaOH solution or modification by silane. A comparative cellulose sample (Figure S1), prepared via swelling in NaOH only, showed that the particles become much smaller with no obvious porous structure, even though the alkaline treatment can change the size, crystal structure and remove some impurity of cellulose [47,48]. These observations indicate that the modification by urea-terminated silane maintains the rough and porous structures of the cellulose particles. 

**Figure 2:**
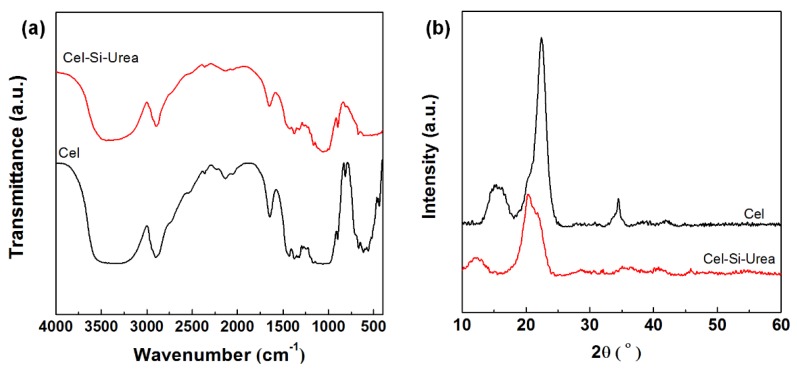
FT–IR spectra (**a**) and XRD patterns (**b**) of the pristine cellulose (Cel) and the urea-terminated silane-modified cellulose (Cel-Si-Urea).

Figure 2 shows the FT–IR spectra (a) and XRD patterns (b) of the pristine and the chemically modified cellulose (Cel-Si-Urea) samples. In Figure 2a, a broad band around 3400 cm^−1^ was observed in the two cellulose samples owing to both asymmetric and symmetric stretching vibrations of –OH groups, which is a typical characteristic band of cellulose. Another special band of cellulose, the C–O–C asymmetric stretching vibration, appeared at 1164 cm^−1^. The band around 2900 cm^−1^ can be attributed to stretching vibration of C–H. The band at 1431 cm^−1^ in the spectrum of pristine cellulose represents bending vibration of C–H [49,50]. The similar band for Cel-Si-Urea is shifted to 1420 cm^−1^ because of the stretching of C–N generally located at 1420–1400 cm^−1^. A strong band at 1640 cm^−1^ may be attributed to the absorbed water, which is shifted to 1650 cm^−1^ for Cel-Si-Urea due to the stretching vibration of C=O. Thus, modification by urea-terminated silane was confirmed. 

Figure 2b shows the XRD patterns of the pristine and modified cellulose samples. The characteristic peaks of pristine cellulose are located around 15.6° and 22.4° [51], corresponding to the diffraction pattern of cellulose I. For the decorated cellulose, two peaks at 12° and 20° were observed, in accordance with the characteristic peaks of cellulose II. From previous studies, the treatment by NaOH always results in crystal transformation from cellulose I to cellulose II. These results also confirm the effect of swelling in NaOH. The degree of crystallinity of the cellulose samples was calculated using Jade 6.0 (MDI, Livermore, CA, USA), the value of which was 32.37% for pristine cellulose and 22.33% for the decorated cellulose. These values indicate that the physical and chemical treatments not only change the crystal form but also reduce the crystallinity of cellulose. 

**Figure 3:**
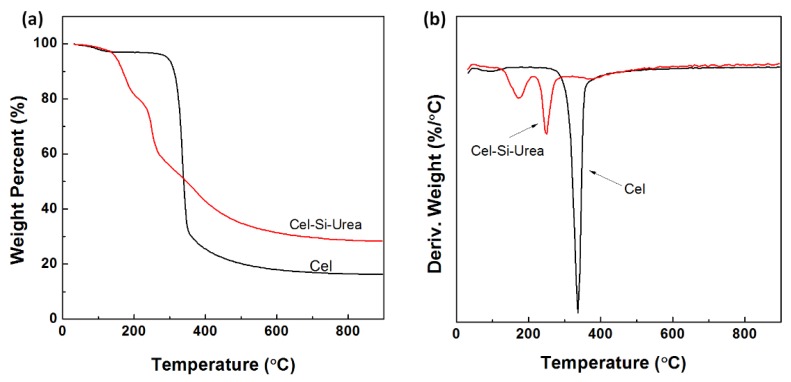
Thermogravimetric analysis (TGA) (**a**) and derivative thermogravimetric analysis (DTG) (**b**) curves of the pristine cellulose (Cel) and the urea-terminated silane-modified cellulose (Cel-Si-Urea).

The thermogravimetric analysis (TGA) and derivative thermogravimetric analysis (DTG) curves of the cellulose samples are shown in Figure 3. In Figure 3a, the pristine cellulose has a weight loss before reaching 100 °C, which is caused by the minute amount of absorbed water because of the high hydrophilicity of cellulose. There is only one main weight-loss step of almost 70% in the temperature range of 270–450 °C. This change relates to the decomposition of the polymer chains of cellulose. Correspondingly, a sharp DTG peak was observed in Figure 3b. The weight loss before 100 °C, for the silane-decorated cellulose, is negligible. Then, three weight-loss steps are observed in the temperature ranges of 120–220, 220–300 and 300–450 °C, attributed to the decomposition of silane, hydrolyzed cellulose chains and the initial cellulose chains, respectively. Therefore, the thermal decomposition process of the treated cellulose becomes complicated and different from that of the pristine cellulose. 

### 3.2. ER Properties

From the above analysis, it was found that the morphological, chemical and condensed structures of the silane-modified cellulose are different from the pristine cellulose. All these differences are considered to have major effects on the ER properties of cellulose. Two ER fluids based on pristine and modified cellulose were prepared with the same weight percent (15%). Dynamic oscillation tests were used to predict the electric field-induced structures in the ER fluids. Amplitude oscillatory shear was firstly applied to determine the linear viscoelastic (LVE) region of the ER fluids under an applied electric field. 

**Figure 4:**
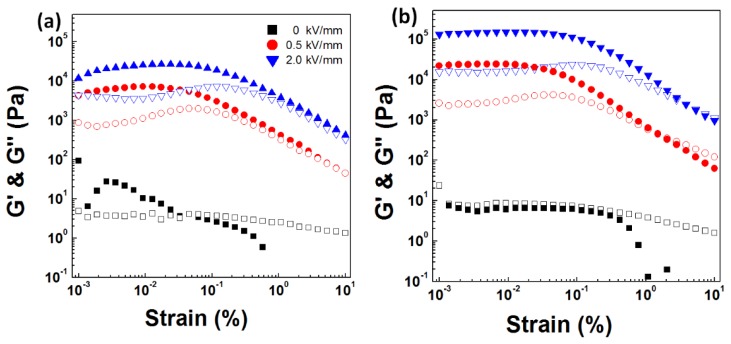
Strain amplitude sweep of the ER fluids at different electric field strengths: (**a**) pristine cellulose; (**b**) urea-terminated silane-modified cellulose (G′: closed symbol; G″: open symbol).

Figure 4 illustrates the storage modulus (G′) and loss modulus (G″) of the two ER fluids measured with amplitude (strain) sweeps ranging from 0.001% to 10% at a constant frequency of 10 rad/s. In Figure 4a, at a zero field, the value of G′ is firstly higher than G″ and then becomes lower than G″ at a very small strain value of 0.03%, indicating that the ER fluid containing rod-like cellulose particles is a viscoelastic solid at low oscillation strain but becomes a viscoelastic liquid when the strain is over a critical value. When an electric field is applied, the cellulose particles form chain-like structures. It was observed that G′ becomes higher than G″ before a critical strain, indicating that the ER fluid has a broad solid-like region at applied electric field. In Figure 4b, both G′ and G″ of the Cel-Si-Urea ER fluid were more stable than those shown by the pristine cellulose ER fluid. When an electric field was applied, there was a stable plateau in G′, which is called the LVE region, caused by the recoverable deformation of the solid-like ER fluid. The value of the modulus and scope of the LVE increased with the electric field. More importantly, both G′ and G″ for the ER fluid of Cel-Si-Urea were higher than those of the pristine cellulose. These data indicate that after being decorated by urea-terminated silane, the particles have stronger interactions than the pristine ones. 

In addition, porous morphology is also considered to have a positive effect on ER performance. This may be due to the porous structure being able to support a larger surface area and thus having a larger interface with the silicone oil to improve the interfacial polarization of the particles. This point of view has been proposed in previous studies [52,53].

**Figure 5:**
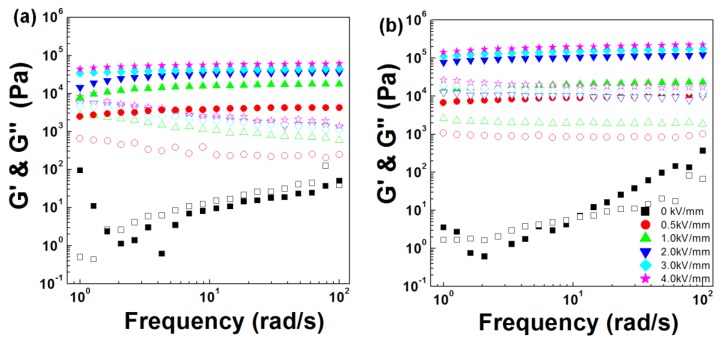
Frequency sweep of the ER fluids at different electric field strengths: (**a**) pristine cellulose; (**b**) urea-terminated silane-modified cellulose (G′: closed symbol; G″: open symbol).

Frequency sweep tests were based on the LVE region measured in the strain amplitude sweeps and conducted in the angular frequency range of 1–100 rad/s with the strain amplitude of 0.003%. Figure 5 shows the G′ and G″ of the two ER fluids measured during the frequency sweeps. In the absence of an electric field, G″ is slightly higher than G′ within a certain frequency range. Both G′ and G″ increase with frequency, confirming the viscoelastic liquid characteristics of the ER fluids. With the application of an electric field, G′ is higher than G″ at all the applied electric field strengths and both remain stable in the measured frequency range. The main difference between the two ER fluids is that the modulus demonstrated by the ER fluid of Cel-Si-Urea (Figure 5b) is higher than those of the ER fluid of pristine cellulose (Figure 5a). This means that the chain structures formed by the decorated cellulose particles are stiffer than those formed by the pristine cellulose, and a higher modulus can be sensed in the oscillatory deformation process. 

**Figure 6:**
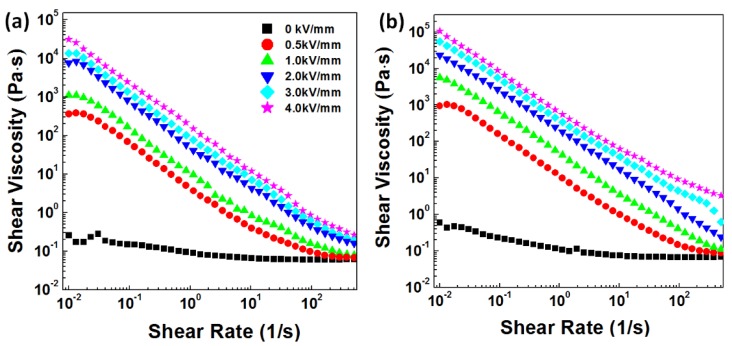
Shear viscosity vs. shear rate at different electric field strengths for the ER fluids of pristine cellulose (**a**) and Cel-Si-Urea (**b**).

**Figure 7:**
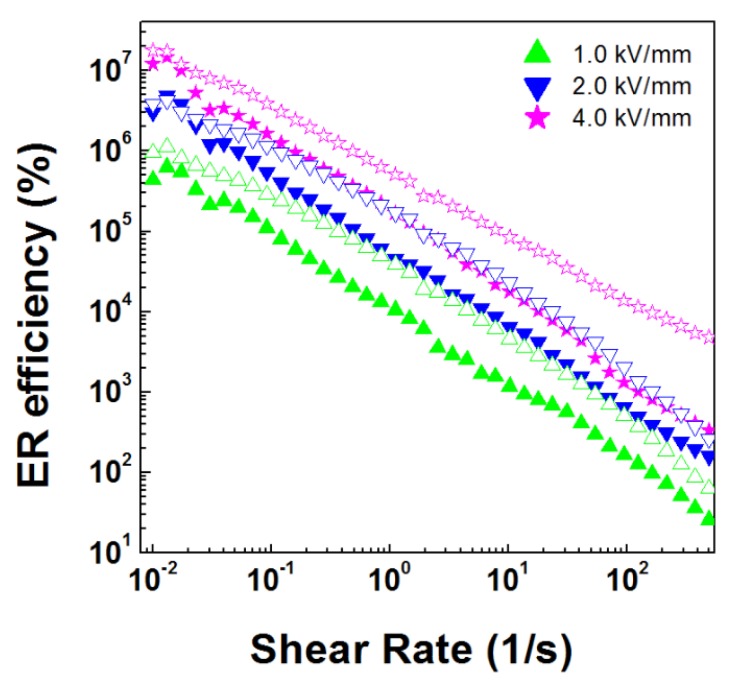
ER efficiency of the two ER fluids: closed symbol for pristine cellulose and open symbol for Cel-Si-Urea.

Shear stress (*τ*) and shear viscosity (*η*), which can be obtained in the steady shear process, are important rheological properties of ER fluids. In this study, both shear stress and shear viscosity curves of the two ER fluids were measured in a controlled shear rate mode ($\dot{\gamma }$ = 0.01–500 1/s). Figure 6 shows the shear viscosity curves of the two ER fluids. At zero electric field, both ER fluids show slight shear-thinning behavior in the low-shear-rate region, and then Newtonian-like behavior with shear-rate-independent shear viscosity in the high-shear-rate region. These data indicate that the cellulose particles have better dispersibility in silicone oil than many other nanostructured ER materials [54,55]. In addition, the values of the shear viscosity of the two ER fluids are similar. This means that the change in particle morphology does not have a significant influence on the zero-field shear viscosity of the ER fluid. When the electric field was applied, obvious shear-thinning behaviors were observed in the two ER fluids. The shear viscosity increases gradually with the electric field strength. More importantly, the Cel-Si-Urea ER fluid (Figure 6b) exhibits higher shear viscosity than the pristine cellulose ER fluid (Figure 6a). For example, when the electric field strength is 4.0 kV/mm and the shear rate is 10^−2^ 1/s, the shear viscosity of the Cel-Si-Urea ER fluid is 10^5^ Pa·s. However, it is only 3 × 10^4^ Pa·s for the pristine cellulose ER fluid. Considering the similar zero-field shear viscosity of the two ER fluids, the Cel-Si-Urea ER also presents higher ER efficiency (*I*), which is defined by the equation as follows:

(4) $$I\left(\%\right)=\frac{\eta_{E}\;-\;\eta_{0}}{\eta_{0}}\;\times \;100,$$

where $\eta_{0}$ and $\eta_{E}$ are the viscosities of the ER fluid at zero and nonzero electric field, respectively. The ER efficiency of the two ER fluids is presented in Figure 7. It is obvious that the Cel-Si-Urea ER fluid shows higher ER efficiency than the pristine cellulose ER fluid in the whole shear rate range. The ER properties of the cellulose particles treated by NaOH only are shown in Figure S2. It is found that at a certain electric field strength, the NaOH-treated cellulose shows a slightly higher shear stress than pristine cellulose, but it is not as high as that of Cel-Si-Urea. Therefore, chemical modification by polar molecules is very important for the enhancement of the ER properties of cellulose. 

**Figure 8:**
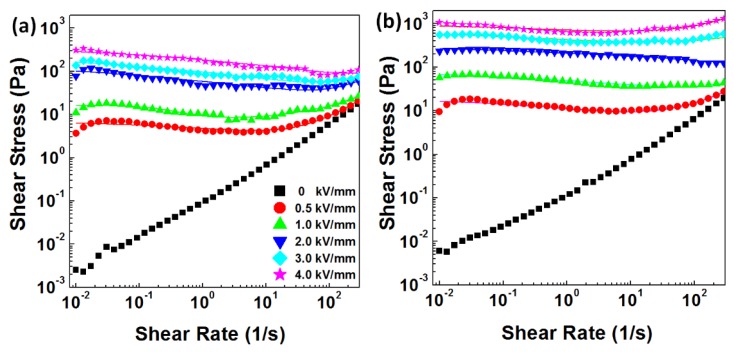
Shear stress vs. shear rate at different electric field strengths for the ER fluids of pristine cellulose (**a**) and Cel-Si-Urea (**b**) (symbols: experimental, lines: fitting of CCJ model to experimental points).

Figure 8 shows the shear stress curves of the ER fluids as a function of shear rate. Without electric field applied, both ER fluids perform like a Newtonian fluid, showing a linear increase in shear stress with shear rate, and both have similar shear stress values. When an electric field is applied, the ER fluids exhibit non-Newtonian behavior with field-dependent yield stresses which can be attributed to the field-induced liquid-to-solid phase transition. In the solid state, yield stress is needed to deform the solid-like structures of the ER fluid. At each electric field strength, the pristine cellulose ER fluid (Figure 8a) shows a slight decrease in shear stress with shear rate. The Cel-Si-Urea ER fluid (Figure 8b) presents similar behavior but with a less obvious reduction of shear stress with shear rate. Similar to the shear viscosity, the Cel-Si-Urea ER fluid also shows higher shear stress than the pristine cellulose ER fluid. The differences in shear stress of the two solid-like ER fluids are mainly due to the robustness of the chains formed by the cellulose particles. The more robust the chains, the higher the shear stress that is needed to deform or destroy the chains under the same shear rate. To analyze the shear stress curves further, a modified constitutive equation for non-Newtonian fluids proposed by Choi et al. [56,57] was used to fit the flow curves. The equation, named the CCJ model, is shown as follows:

(5) $$\tau =\frac{\tau_{y}}{1+{\left(t_{2}\dot{\gamma }\right)}^{\alpha }}+\eta_{\infty }\left(1+\frac{1}{{\left(t_{3}\dot{\gamma }\right)}^{\beta }}\right)\dot{\gamma },$$

where *τ_y_* and *η*_∞_ denote the dynamic yield stress and viscosity at an infinite shear rate, respectively, *t*_2_ and *t*_3_ are time constants, and *α* and *β* are fitting parameters related to the shape of flow curves. The fitting values of the parameters are shown in Table 1. The modified model is able to fit the shear stress curves very well in the measured shear rate range.

**Figure 9:**
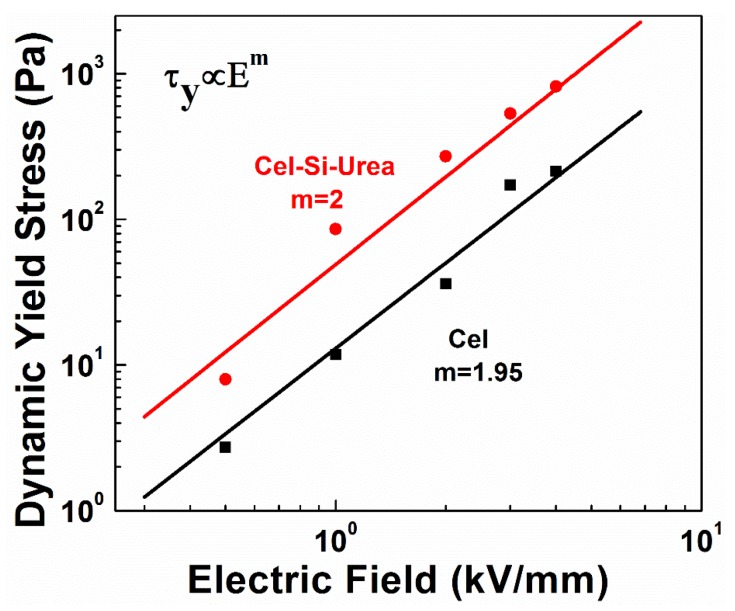
Dynamic yield stress of the ER fluids as a function of electric field strength (symbols: dynamic yield stress obtained from CCJ model, lines: fitting of power-law equation).

The dynamic yield stresses obtained from the above fitting process were re-plotted as a function of electric field strength, as shown in Figure 9. A power-law equation, $\tau_{y}\propto E^{m}$, was used to fit the dynamic yield stress of each ER fluid. The value of the power exponent *m* is in the range of 1.0 to 2.0, according to the characteristics of the particles [58,59]. In this situation, it is 1.85 and 2.0 for the pristine cellulose and Cel-Si-Urea, respectively, indicating that the decorated cellulose has a higher field-dependent increase in dynamic yield stress.

**Figure 10:**
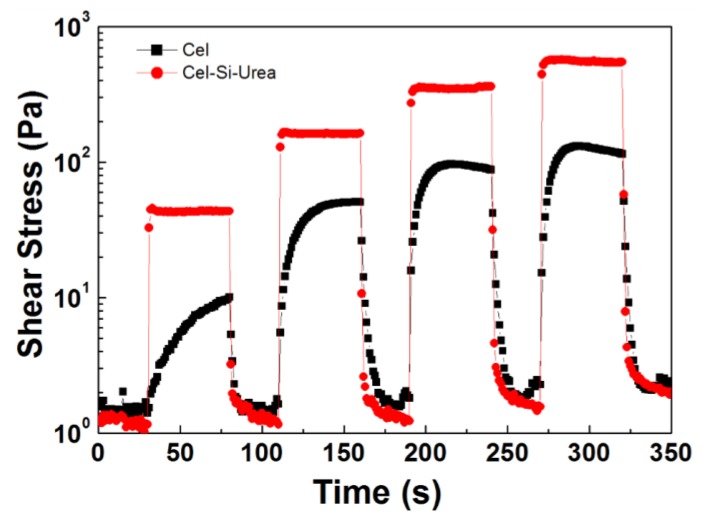
Shear stress of the ER fluids based on pristine cellulose (square) and decorated cellulose (circle) at the square voltage pulse electric field.

Square wave pulse voltage measurements were used to observe the instantaneous response of the ER fluid to an electric field. Figure 10 shows the shear stress of the two ER fluids measured at a fixed shear rate of 1 1/s and a square pulse electric field (0.5–4 kV/mm). Once the electric field is applied, the shear stress of the Cel-Si-Urea ER fluid jumps rapidly to a higher value and reaches a plateau. However, the shear stress of the pristine cellulose ER fluid grows gradually until reaching a critical value at an applied electric field. This implies that the Cel-Si-Urea particles can respond to the electric field rapidly and form stable chain structures within a very short time. In contrast, the pristine cellulose needs a period of time to arrange the chain structures. It was observed that the Cel-Si-Urea ER fluid shows a higher shear stress than the pristine cellulose ER fluid at the same electric field strength. At the switched-off point, both ER fluids cannot immediately transform from a solid-like state to liquid-like state. The reason for the delayed transition is that when the electric field is removed, the polarized particles need a longer time to relax to their original unpolarized states with mechanical shear as the only driving force. Therefore, when the square wave electric field is applied, the ER fluids show different response behaviors [60]. Nevertheless, the Cel-Si-Urea ER fluid still responds more rapidly than the pristine cellulose ER fluid. 

**Figure 11:**
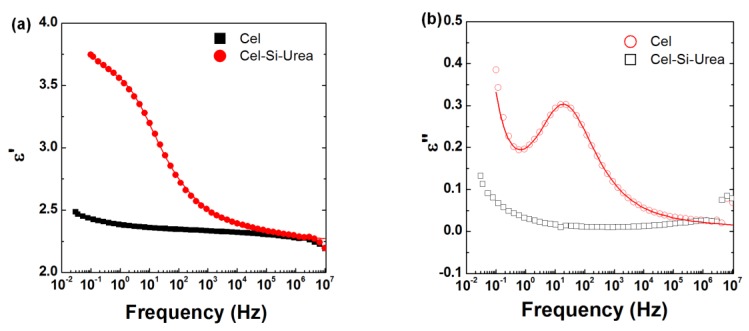
Dielectric spectra of the ER fluids: dielectric constant *ε*′ (**a**) and dielectric loss factor *ε*″ (**b**), Cel represents the pristine cellulose ER fluid and Cel-Si-Urea is for the urea-terminated silane-modified cellulose ER fluid. The solid lines were fitted using the modified HN equation.

The rheological properties of the ER fluids under electric fields are closely related to their dielectric characteristics, which indicate the field-induced polarization dynamics of the ER fluids. In this study, the dielectric spectra of the two ER fluids were measured by a broadband dielectric spectrometer at a frequency range of 0.03–10^7^ Hz. In Figure 11, the dielectric constant (*ε*′) and the dielectric loss factor (*ε*″) of the two ER fluids are presented and analyzed by the Havriliak–Negami (HN) equation [61,62], modified by a conductivity term and an electrode polarization term [63]:

(6) $$\varepsilon^{*}\left(\omega \right)=\varepsilon^{\prime }+i\varepsilon^{\prime \prime }=\varepsilon \prime_{\infty }+\frac{\Delta \varepsilon \prime }{{\left(1+{\left(i\omega \tau \right)}^{\alpha }\right)}^{\gamma }}+\frac{\sigma_{dc}}{i\varepsilon \prime_{0}\omega }+A\omega^{-n},$$

where Δε′ = ε′_∞_ − ε′_0_ is the dielectric strength and represents the polarizability of the dispersed phase, *ε*′_0_ and *ε*′_∞_ are the dielectric constants at low and high frequency, respectively, *τ* = 1/(2π*f*_max_) denotes the dielectric relaxation time, *α* and *γ* are the profile shape factors of the relaxation dispersion, *σ*_dc_ represents the dc-conductivity at low frequency, and *n* is associated with the slope at high frequency. The parameters obtained from fitting by the HN equation are shown in Table 2. 

In Figure 11a, the Cel-Si-Urea ER fluid shows higher *ε*′ than the pristine cellulose ER fluid. Figure 11b represents the *ε*″ spectra of the ER fluids. There is an obvious dielectric relaxation for the Cel-Si-Urea ER fluid and the pre-increase in *ε*″ is caused by dc-conductivity. For the pristine cellulose ER fluid, no dielectric relaxation appears in the measured frequency range. In a previous study, it was confirmed that both Δ*ε*′ and *τ* are crucial parameters in determining ER properties of an ER fluid [61]. Therefore, the larger Δ*ε*′ and obvious dielectric loss for the Cel-Si-Urea ER fluid are consistent with the excellent ER performance. The larger Δ*ε*′ of the Cel-Si-Urea ER fluid may be associated with the porous morphology of the treated cellulose particles, which could result in a larger interfacial polarizability. In addition, the urea polar molecule is considered to contribute to the polarization of cellulose. 

## 4. Conclusions

The pristine cellulose particles were modified using a urea-terminated silane, 1-(3-(trimethoxysilyl) propyl) urea, before which the particles were pretreated in NaOH solution at low temperature. The morphology and crystal structures of the cellulose particles dramatically changed after physical swelling and chemical modification. Compared to the ER effects of the pristine cellulose, the decorated cellulose showed very similar rheological properties without the stimulus of an electric field, but presented higher dynamic yield stress and dynamic modulus when an electric field was applied, and higher sensitivity to electric field. Dielectric analysis indicated that the silane-decorated cellulose with porous morphology had higher dielectric strength with an obvious dielectric loss which was not observed in the pristine cellulose. It confirmed that the double effect of silane modification, maintenance of the porous structure and introduction of highly polar groups, had a positive effect on the ER properties of cellulose particles. The modification by silane could also reduce the sensitivity of cellulose to water, which is crucial for the commercial application of cellulose-based ER fluid.

## Figures and Tables

**Table 1:** Parameters of CCJ model (Equation (5)) obtained from the fitting of shear stress curves of the ER fluids.

Sample	E (kV/mm)	0.5	1.0	2.0	3.0	4.0
Cel-Si-Urea	*τ_y_*	7.99	85.7	271	532	817
	*α*	1.14	0.17	0.31	0.32	0.79
	*β*	0.98	0.30	0.50	0.24	0.90
	*t*_2_	1.49	0.05	0.01	0.01	8.37
	*t*_3_	0.01	0.47	0.06	0.04	0.002
	*η*_∞_	0.07	0.08	0.09	0.13	1.49
Pristine cellulose	*τ_y_*	2.72	11.7	36.2	173	214
	*α*	1.81	1.25	1.78	0.28	0.36
	*β*	1.00	0.90	1.09	0.18	1.44
	*t*_2_	2.93	2.86	10.4	0.30	0.07
	*t*_3_	0.02	0.004	0.002	0.30	0.04
	*η*_∞_	0.06	0.52	0.08	0.09	0.17

**Table 2:** Parameters in Equation (6) fitted from the dielectric spectra of the Cel-Si-Urea ER fluid.

**Cel-Si-Urea**	**Δ*ε’***	***ε’*_0_**	***ε’*_∞_**	***α***	***γ***	***τ***	***σ***	***A***	***n***
	1.38	3.63	2.25	0.65	0.82	0.0098	0.048	0.19	−0.1

## Supplementary Materials

The following are available online at http://www.mdpi.com/2073-4360/10/8/867/s1, Figure S1: SEM of physically treated cellulose particles in NaOH solution, Figure S2: Shear stress and shear viscosity curves of the ER fluid containing physically treated cellulose in NaOH solution.
