# Supplementary material for: Enhanced Electrorheological Response of Cellulose: A Double Effect of Modification by Urea-Terminated Silane
Source: Polymers (Basel). 2018 Aug 4;10(8):867. doi: 10.3390/polym10080867 (PMC6403862; doi:10.3390/polym10080867)
Supplement: Supplementary file 1 [file polymers-10-00867-s001.pdf]

Supplementary Materials

Enhanced Electrorheological Response of Cellulose: A Double Effect of Modification by Urea-terminated Silane

Zhao Liu [1], Panpan Chen [1], Xiao Jin [1], Li-Min Wang [1], Ying Dan Liu [1,*] and Hyoung Jin Choi [2,*]

[1] State Key Lab of Metastable Materials Science and Technology, College of Materials Science and Engineering, Yanshan University, Qinhuangdao 066004, Hebei, China; liuzhao0120@sina.cn (Z.L.); 15032375221@139.com (P.C.); jinxiao94315@163.com (X.J.); limin_wang@ysu.edu.cn (L.-M.W.)

[2] Department of Polymer Science and Engineering, Inha University, Incheon 402751, Korea

* Correspondence: ydliu@ysu.edu.cn (Y.D.L.); hjchoi@inha.ac.kr (H.J.C.)

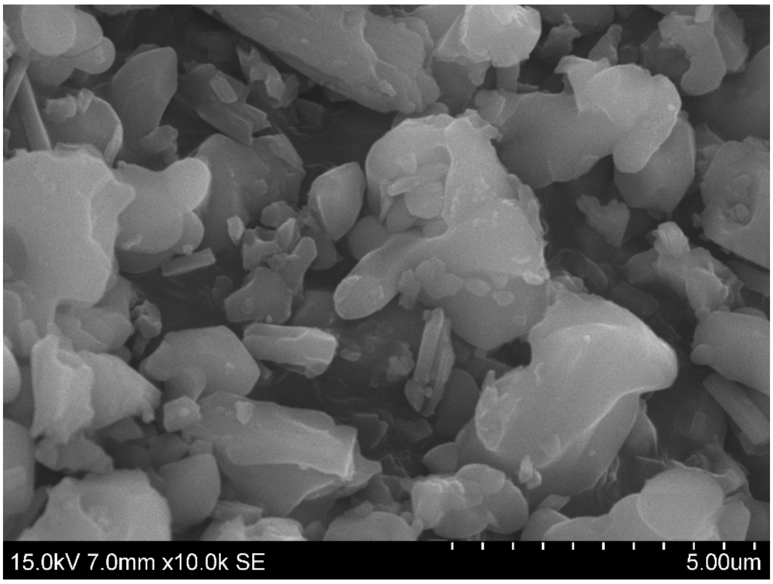

Figure S1. SEM of physically treated cellulose particles in NaOH solution.

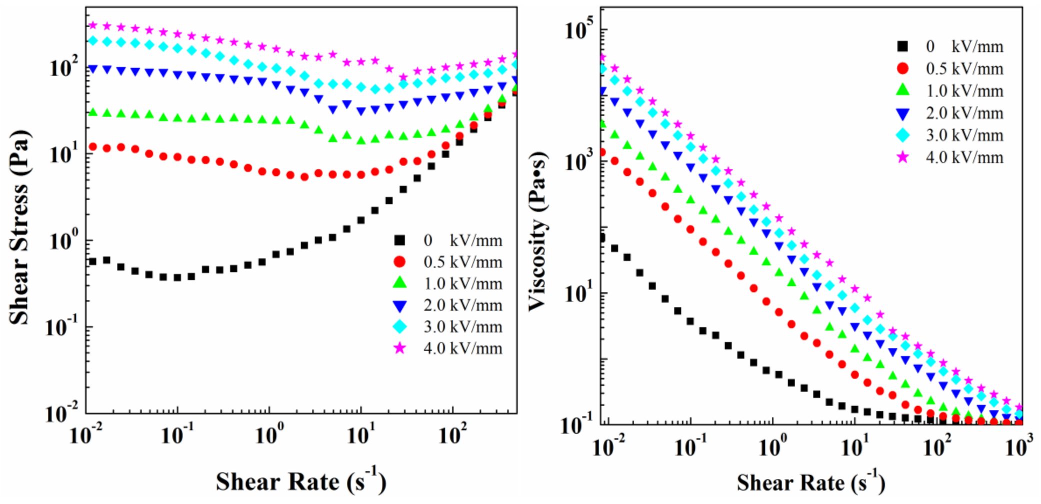

Figure S2. Shear stress and shear viscosity curves of the ER fluid containing physically treated cellulose in NaOH solution. .
